# Canine Hemangioblastoma: Case Series and Literature Review

**DOI:** 10.3390/ani15203010

**Published:** 2025-10-16

**Authors:** Çağla Aytaş, Alberto Cauduro, Cristian Falzone, Stefania Gianni, Anna Tomba, Carlo Cantile

**Affiliations:** 1 Dipartimento di Scienze Veterinarie, University of Pisa, Viale delle Piagge n. 2, 56124 Pisa, Italy; cagla.aytas@phd.unipi.it; 2 Clinica Veterinaria Neurovet, Via Maestri del Lavoro n. 29, 20025 Legnano, Italy; a.cauduro@neurovet.it; 3 Diagnostica Piccoli Animali s.r.l, Clinica Veterinaria Pedrani, Via Caldierino n. 13, 36030 Zugliano, Italy; crisfalz77@gmail.com; 4 Clinica Veterinaria Tibaldi, Via Pezzotti n. 2, 20141 Milan, Italy; stefania.neurologia@gmail.com; 5 Norad, Centro Neurologico Veterinario, Via Arnaldo Agnelli n. 21, 21017 Gallarate, Italy; annatomba@yahoo.it

**Keywords:** hemangioblastoma, spinal cord, peripheral nerve, neuroimaging, neuropathology, neuro-oncology, immunohistochemistry, dog

## Abstract

**Simple Summary:**

Canine hemangioblastoma is a rare tumor of the central nervous system, predominantly affecting the spinal cord. The aim of this study was to determine the clinical signs, specific location, neuroradiological features, pathological patterns, immunophenotype, and follow-up by evaluating six cases from our archive and comparing the results with the available literature. Canine hemangioblastoma shows morphological and immunohistochemical features comparable to the human counterpart and surgery may be effective in cases of intradural-extramedullary and peripheral nerve locations of the tumor, as in humans.

**Abstract:**

Human hemangioblastoma is a benign, slow-growing, highly vascular neoplasm. The tumor most commonly arises in the cerebral hemispheres and cerebellum, where it is more frequently observed in patients with von Hippel–Lindau disease. In veterinary medicine, hemangioblastoma has only been described in the central nervous system of dogs and in the skin of lambs. Our study aimed to characterize the clinical and neuropathological features of five cases of canine spinal cord hemangioblastoma and one case of sciatic nerve localization, and to compare these results with those reported in the veterinary literature. Diagnoses were achieved by neurological examination, neuroimaging, surgery or post-mortem examination, histopathology, and immunohistochemistry. All tumors were composed of numerous, haphazardly arranged capillaries lined by plump endothelium and interstitial fusiform to stellate stromal cells. Immunohistochemically, the stromal cells were strongly immunolabeled with NSE and carbonic anhydrase IX and were negative for von Willebrand factor VIII and inhibin-α. Canine hemangioblastoma exhibits morphological and immunohistochemical features comparable to the human counterpart, although the latter is mostly positive for inhibin-α. Surgery may be effective in cases of intradural-extramedullary and peripheral nerve locations, as in humans. This is the first report of peripheral nerve hemangioblastoma in animals.

## 1. Introduction

Hemangioblastomas (HBs) are rare, highly vascularized tumors that primarily affect the central nervous system (CNS). In humans they account for 1.5–2.5% of all intracranial tumors [1]. HBs are classified as WHO grade I and in approximately 70% of cases can occur sporadically in every region of the CNS as solitary lesions. In the remaining 30%, they represent a manifestation of von Hippel–Lindau (VHL) disease and tend to develop as multiple HBs, as well as extraneural tumors, including clear cell renal cell carcinoma, pheochromocytoma, pancreatic neuroendocrine, and endolymphatic sac tumors [2]. In this latter cohort of patients, the cerebellum is predominantly affected, whereas less frequent locations are the supratentorial compartment, retina, brainstem, spinal cord, and spinal nerve roots [3]. HBs in the spinal cord account for as much as 3% of all spinal cord tumors. HBs outside the CNS are exceptional and have been reported in the liver, lungs, kidneys, bones, stomach, pancreas, adrenal glands, bladder, tongue, skin, and peripheral nerves [4]. VHL disease results from the allelic loss or inactivation of the *VHL* tumor suppressor gene. Somatic *VHL* loss of function is also demonstrated in almost 80% of sporadic cases [5]. Furthermore, methylation profiling of HBs revealed two molecular subgroups with distinct methylation components and differences in cytogenetic profiles [6].

The first report of HB of the canine CNS appeared in the veterinary literature in 2003 [7]. Up to now, HB has been found in the CNS of 11 dogs [8,9,10,11,12,13], including 4 cases that have been retrospectively reevaluated [14,15]. Only one case of extraneural HB has been described in the skin of a lamb [16]. Since VHL disease is not recognized in animals, all recorded HB cases can be classified as spontaneous tumors.

In this study, we describe the clinico-pathological features of 5 cases of canine spinal HB and one case of peripheral nerve involvement. Since most of the existing literature is represented by case reports with fragmented information, this retrospective study aimed to determine the clinical signs, specific location, neuroradiological features, pathological patterns, immunophenotype, and follow-up by evaluating the cases in our archive and comparing the results with the available literature.

## 2. Materials and Methods

### 2.1. Caseload

Six cases of central and peripheral nervous system HB diagnosed between 2007 and 2024 were retrieved from the archive of the Neuropathology Laboratory of the Department of Veterinary Sciences of Pisa University. Specimens were obtained either from surgical biopsy or post-mortem examination, routinely formalin-fixed, paraffin wax-embedded, and processed for histology. Complete medical records included information on breed, gender, age at presentation, physical and neurological examination findings, neurolocalization of the lesion, magnetic resonance imaging (MRI), complete blood cell count, serum biochemical profile, treatment, and outcome.

### 2.2. Neuroimaging

MRI was performed in all cases and the protocol consisted of sagittal, transverse, and dorsal T2-weighted imaging (T2WI) and T1-weighted imaging (T1WI), with the latter repeated after contrast medium administration (0.2 mmol/kg IV gadolinium). When present, additional sequences, such as short-tau inversion recovery (STIR) and fat saturation (Fat Sat) imaging were also reviewed.

### 2.3. Histopathology and Immunohistochemistry

Four-µm tissue sections were stained with hematoxylin and eosin (H&E), Luxol Fast Blue (LFB), toluidine blue, and Goldner trichrome staining. For each tumor, the number of cells (cellularity), capillaries, inflammatory cells, cytoplasmic vacuoles, and the amount of fibrotic tissue were considered. All tumor samples were assigned to a category showing an orderly progression in severity, from absent to marked, of their respective morphological features. Two authors (ÇA and CC) independently assessed the characteristics and extent of the tumors, and a consensus diagnosis of immunoreactivity was made.

Immunohistochemistry (IHC) was performed using the avidin–biotin–peroxidase complex method and epitope retrieval was carried out at 120 °C in a pressure cooker for 3 min with a Tris/EDTA buffer pH 9.0. Primary antibodies against the following antigens were used: neuron specific enolase (NSE), vimentin (VIM), von Willebrand factor VIII (vWF), glial fibrillary acidic protein (GFAP), inhibin-α (INHα), carbonic anhydrase IX (CAIX), and Ki-67. Details of the primary antibodies are provided in Table 1. The EnVision Plus System-HRP (3,3′-diaminobenzidine, Dako) was used to detect antibody binding. Negative controls were obtained by omitting the primary antibody. Positive controls for INHα and CAIX were canine ovary and intestine, respectively. Normal canine nervous tissue was used as positive controls for NSE, vWF, VIM, and GFAP primary antibodies.

## 3. Results

### 3.1. Clinical and Imaging Findings

Signalment including breed, gender, age, lesion location, tissue sample origin (post-mortem examination or surgical biopsy), and outcome are summarized in Table 2.

Dogs affected by HB had a mean age of 8.7 ± 1.6 years, ranging from 6 to 11 years. There were equal numbers of males and females. Two dogs were Labrador Retrievers and there was one of the following breeds: Boxer, Beagle, Yorkshire Terrier, and a mixed breed.

Blood tests yielded unremarkable results in all dogs. Neurological signs at presentation were progressive paraparesis (dog 1), left hemiparesis for 1 year (dog 2), progressive tetraparesis with cervical pain over the last 2 months (dog 3), monoparesis of the right pelvic limb for 1 year (dog 4), and progressive paraparesis to paraplegia with the presence of deep pain perception (dog 5). Dog 6 showed lameness of the left pelvic limb with mild proprioceptive deficit and absence of the flexor reflex, in association with atrophy and pain of the thigh muscles, slowly progressive in about 2 months.

**Figure 1:**
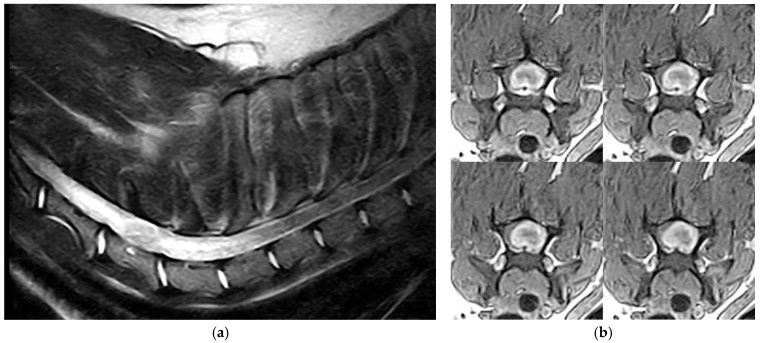

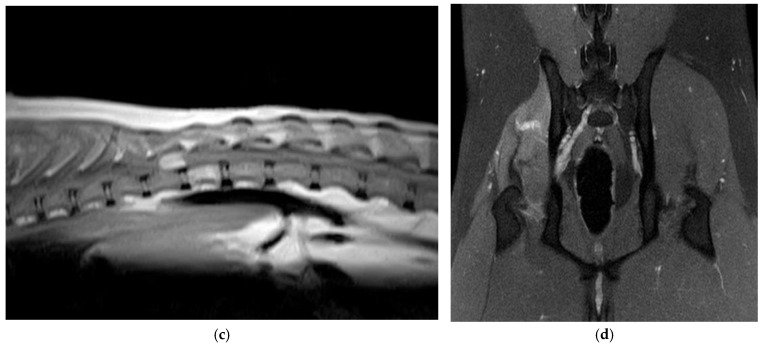
Magnetic resonance imaging of hemangioblastoma. (**a**,**b**) Dog 2, cervical spinal cord. (**a**) Intramedullary lesion with heterogeneous hyperintensity on T2WI. (**b**) Strong heterogeneous post-contrast T1WI enhancement of the lesion. (**c**) Dog 5, thoracolumbar spinal cord. Post-contrast T1WI shows homogeneous enhancement of the intradural-extramedullary lesion with severe spinal cord compression. (**d**) Dog 6, hyperintensity of the right sciatic nerve associated with muscle atrophy (Post-contrast T1WI Fat Sat).

Five of six tumors (dogs 1–5) were in the spinal cord from the cervical to the thoracolumbar segment (C2-T12). In 2 dogs, the tumor location was supposed to be intramedullary because of swelling of the spinal cord without evidence of deviation. In 3 dogs a focal enlargement of the subarachnoid space immediately rostrally or caudally, suggested an intradural-extramedullary location of the mass. In dog 6, the tumor affected the right sciatic nerve. In 5 dogs (1–5), T2WI showed a well-circumscribed, round/oval-shaped, iso- to mildly hyperintense spinal cord mass largely occupying the transverse section of the spinal cord, accompanied by marked edema extending rostrally and caudally to the mass (Figure 1a). Post-contrast T1WI showed marked, homogeneous contrast enhancement of the mass with strong ring enhancement (Figure 1b,c). Dog 6 showed marked swelling of the right sciatic nerve with a transverse size of approximately 6 mm at the point of maximum thickness, ventrocaudal to the sacroiliac joint. The lesion continued proximally at the paravertebral, foraminal, and epidural levels, involving the ipsilateral L6, L7, and S1 nerve roots, in the absence of spinal cord invasion. The nerve structures were hyperintense on STIR images and showed moderate contrast enhancement on T1-Fat Sat images. Atrophy of the middle gluteus and adductor muscles, and partly of the semitendinosus and semimembranosus muscles, was also detected (Figure 1d).

Dog 1 was euthanized due to a poor prognosis. Dogs 2 to 6 underwent surgery with complete resection of the lesions. In dog 2, a durotomy was performed to visualize the entire mass, and a myelotomy was required to remove the lesion en bloc under microscopic surgery. The left C6 and C7 nerve roots were involved and partially removed along with the mass (Video S1). Follow-up evaluations were conducted 1 to 3 years after the surgery, confirming the absence of recurrence in 4 cases and recurrence of the tumor after 2 years in dog 4. Dog 5 improved from paraplegia to ambulatory paraparesis with periodic worsening successfully treated with steroids. The owners of dog 4 declined any further investigation and elected euthanasia; a post-mortem examination was not performed. Dog 6 showed a mild degree of lameness after the removal of the sciatic nerve but did not show any signs of clinical relapse at the time of writing.

### 3.2. Pathological Findings

**Figure 2:**
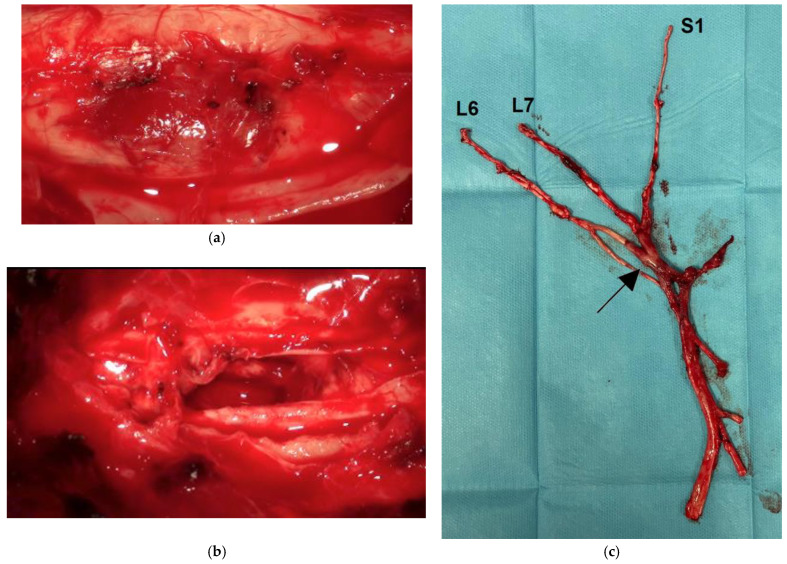
Gross morphology of spinal cord hemangioblastoma. Dog 2. (**a**) Appearance of the tumor after durotomy; (**b**) myelotomy and en bloc resection of the tumor. (**c**) Sciatic nerve hemangioblastoma. Dog 6. Complete resection of the sciatic nerve including the L6-S1 roots. The nerve shows a dark red and thick segment with intense vascularity (arrow).

The macroscopic features of the spinal cord tumors were similar and characterized by well-circumscribed, soft, reddish-purple nodular masses, ranging in diameter from 5 to 11 mm (Figure 2a,b). The tumors markedly compressed the spinal cord and were associated with extensive peritumoral edema. In dog 6, the sciatic nerve was enlarged, firm, and dark red, particularly in the segment ventrocaudal to the sacroiliac joint (Figure 2c).

**Figure 3:**
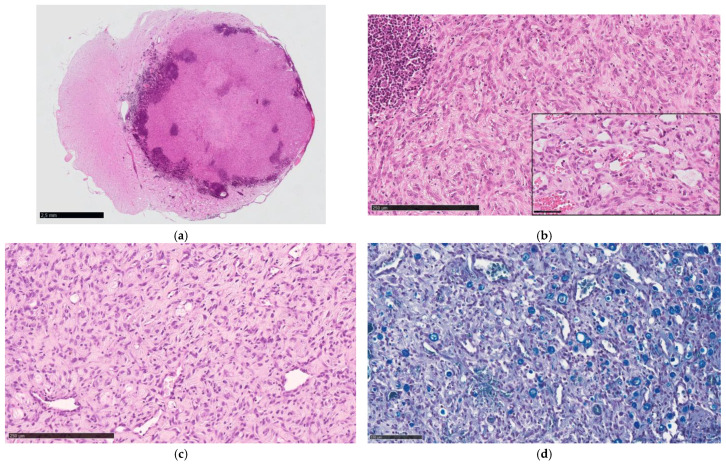
Histomorphology of hemangioblastoma. (**a**) Thoracic (T9 level) spinal cord hemangioblastoma. Dog 1: Subgross section showing extensive inflammatory aggregates surrounding the tumor and a fibrotic central area. Note midline shifting and peritumoral edema of the neuroparenchyma. Bar = 2.5 mm. (**b**) Spinal cord hemangioblastoma. Dog 2: highly cellular tumor composed of numerous closely packed capillaries separated by large pleomorphic stromal cells. An inflammatory aggregate is in the upper left corner (H&E). Bar = 250 µm. Inset: Detail of capillaries and stromal cells (H&E). Bar = 50 µm. (**c**) Sciatic nerve hemangioblastoma. Dog 6: Capillaries and residual nerve fibers are separated by interstitial stromal cells. (H&E). Bar = 250 µm. (**d**) Sciatic nerve hemangioblastoma. Dog 6: Residual nerve fibers show different degrees of axonal and myelin degeneration (LFB). Bar = 100 µm.

Histologically, all tumors were biphasic, composed of numerous, haphazardly arranged capillaries lined by plump endothelium and interstitial fusiform to stellate stromal cells with slightly eosinophilic cytoplasm, occasionally containing small lipid vacuoles. The nuclei were large and round to oval, with open chromatin and a distinct nucleolus. Frequently, surrounding the capillaries were moderate to prominent perivascular aggregates composed of plasma cells and fewer lymphocytes (Figure 3a,b). No mast cells were observed. Varying amounts of fibrotic tissue were detected in the deeper areas of the tumor, highlighted by Goldner staining. The surrounding spinal parenchyma was not infiltrated by the neoplastic cells but was compressed, edematous, and gliotic, with myelin vacuolization and occasional axonal swelling. The sciatic nerve tumor showed identical features with a looser appearance of the interstitial tissue and wider capillaries (Figure 3c). Within the neoplastic proliferation, entrapped nerve fibers showed different degrees of myelin and axonal degeneration (Figure 3d). No neoplastic invasion was observed at the proximal stumps of L6, L7, and S1. The distal stump shows extensive endoneural fibrosis associated with Wallerian degeneration of residual nerve fibers. The morphological features of each tumor are summarized in Table 3.

**Figure 4:**
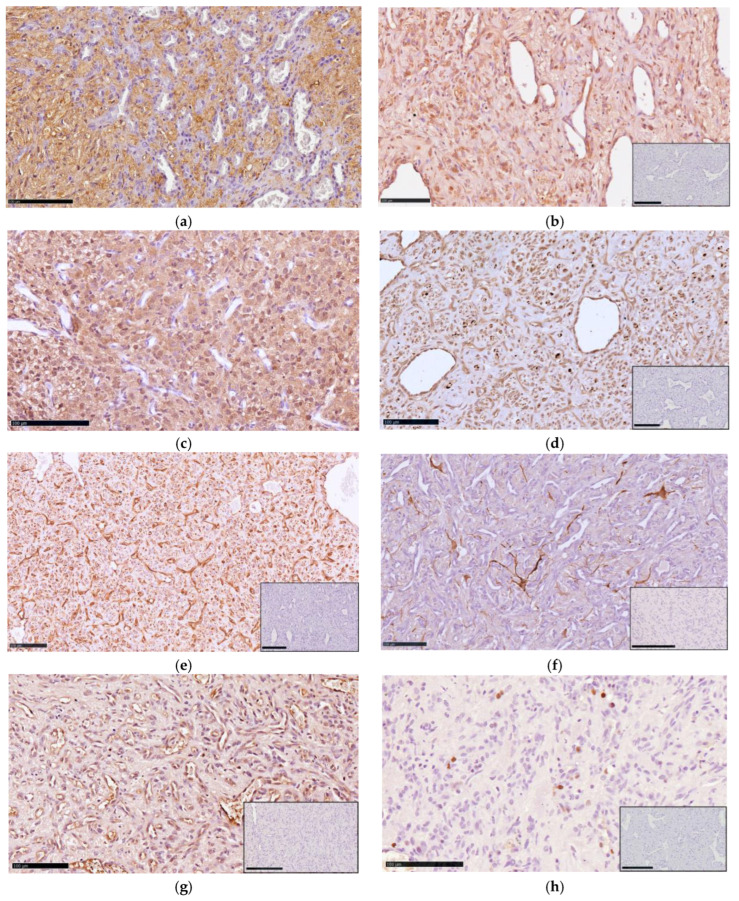
Immunohistochemical profile of hemangioblastoma. Spinal cord (**a**,**c**,**e**,**f**–**h**) and sciatic nerve (**b**,**d**) hemangioblastoma. (**a**,**b**) Dogs 2 and 6. Stromal cells strongly express NSE (NSE IHC); (**c**,**d**) Dogs 4 and 6. Diffuse immunoreactivity of the stromal cells with CAIX (CAIX IHC); (**e**) Dog 6. Marked vimentin expression of both endothelial and stromal cells (VIM IHC); (**f**) Dog 1. Scattered cells within the tumor are immunolabeled with GFAP (GFAP IHC); (**g**) Dog 3. Endothelial cells are consistently immunolabeled with vWF (vWF IHC); (**h**) Dog 6. Nuclear expression of Ki-67 (Ki-67 IHC). Bar = 100 µm. Insets show negative controls for each marker (Bar = 250 µm).

The stromal cells were strongly immunolabeled with NSE (Figure 4a,b), CAIX (Figure 4c,d) and vimentin (Figure 4e). Scattered stromal cells also expressed GFAP (Figure 4f). Stromal cells were consistently negative for vWF and INHα (Figure S1). Endothelial cells were immunolabeled with vWF (Figure 4g) and vimentin. The Ki-67 nuclear labeling index ranged from 5% to 20% (Figure 4h).

## 4. Discussion

HB is a rare canine tumor that, in most cases, affects the spinal cord. Intracranial location is exceptional, and involvement of the peripheral nerve was recorded in only one dog in our series. A summary of the clinical features of canine HBs reported in the literature is shown in Table 4.

Two additional cases from the Cornell University archive (Ithaca, NY, USA) were identified as spinal cord HB, previously diagnosed as capillary hemangioma [15]. Signalment, clinical data, and tumor location were not reported.

No significant difference was found in the age of onset of clinical signs when comparing our cases with those reported in the literature. Taking together all cases, the age of most dogs ranged from 6 to 11 years, but 3 young dogs of 2 and 3 years were also affected. No gender prevalence was noted. Different canine breeds were represented with 2 recorded cases for each of the following breeds: Pointer, Yorkshire terrier, and Labrador Retriever.

Fourteen out of 17 (82.3%) tumors were in the spinal cord (predominantly intramedullary), 2/17 (11.8%) were intracranial, and 1/17 (5.9%) involved the peripheral nerve. In most cases, the clinical signs were nonspecific and directly correlated with the affected spinal segment. In cervical tumors, common findings included chronic and progressive tetraparesis and ataxia, sometimes associated with neck pain and rigidity; in thoracolumbar tumors, pelvic limb stiffness, paraparesis, decreased spinal reflexes, and altered proprioception were observed. Intracranial location was associated with seizures, depression, circling, head tilt, and ataxia. Sciatic nerve involvement was related to lameness and neurogenic muscle atrophy.

Peripheral nerve HB is exceedingly rare in humans, with only 5 published cases [17]. Affected nerves were radial, median, phrenic, sciatic, and ulnar. In 4 cases, no recurrence was observed after total resection. Histologically and immunohistochemically, the tumors were indistinguishable from CNS HB.

On MRI, canine spinal cord HBs tended to appear from iso- to inhomogeneously hyperintense on T2WI and post-contrast T1WI, with strong contrast enhancement and a tendency toward a ring-like pattern, consistent with pronounced peripheral vascularization and central fibrosis. The MRI characteristics were nonspecific, and similar findings can readily occur with meningioma, nephroblastoma, histiocytic sarcoma, and lymphoma [9,11], as well as with non-neoplastic lesions, such as granulomas. In some cases, the precise location of the tumor, whether completely intradural-extramedullary or intramedullary, was not readily determined, often due to the large size of the mass. However, during surgery or post-mortem examination, a dissection plane could be easily identified [11], and no tumor infiltration of the neuroparenchyma was observed [7,10]. Intracranial HBs had similar MRI signals and were characterized by iso- to hypointense T1WI with strong contrast enhancement and isointense to hyperintense T2WI. The lesions were accompanied by peritumoral edema and hemorrhagic areas. In human HBs, peritumoral cysts are frequently associated with symptomatic tumors [18]. Rarely, intraparenchymal, subarachnoid, or ventricular hemorrhage may be observed [18,19]. No cyst formation is described in canine HBs.

Grossly, spinal cord HBs typically appeared as well-circumscribed round nodules of 4 to 15 mm in diameter, usually reddish, and accompanied by prominent and congested meningeal vessels and spinal cord edema [7,10,11,12]. Intracranial tumors appeared to originate from meninges in one case [9] and as a dark red intraparenchymal brainstem mass in another case [13].

On histological sections, HBs demonstrated two main components: neoplastic large stromal cells with variable cytoplasmic vacuolation and abundant reactive capillary vessels. The stromal cells are considered the neoplastic element, driving tumor growth and neovascularization. Indeed, the recognition of multiple angiogenic signaling pathways may synergistically contribute to the abundant vascularization [20]. Two histologic variants have been recognized in human HBs: in the reticular variant, the vascular network is predominant over stromal cells, whereas in the less common cellular variant, the stromal cells are more abundant, arranged in sheaths, and may reveal solid epithelioid aggregates possibly associated with extramedullary hematopoiesis [21]. All reported cases of canine HB can be classified as the reticular variant. However, some morphologic differences should be noted between the canine tumor and human HB. The stromal cells in the canine tumor do not contain an appreciable amount of lipid, which is characteristic of the human counterpart. In humans, mast cells are commonly distributed rather uniformly throughout the tumor [22], whereas extensive peritumoral inflammation and central desmoplasia are not common features.

Differential diagnosis included other low-grade primary tumors or malformations with vascular components that have been occasionally reported in the canine spinal cord [15]. A cavernous angioma has been described and consists of numerous dilated, tightly packed, thin-walled vascular channels without intervening tissue [23]. A spinal cord vascular hamartoma was characterized by proliferation of thick-walled vessels of varying caliber without intervening neuroparenchyma [14]. Another rare condition, presumably of malformative origin, is meningioangiomatosis. This entity has been reported in the cervical [24] and thoracolumbar [25] spinal cord of young dogs. The lesion is composed of the perivascular spread of a mixed population of meningothelial and fibroblastic cells that invade the nervous tissue from the leptomeninges. Because HB may present as masses attached to the dura, the differential diagnosis with hypervascular angiomatous meningioma should also be considered. This variant is characterized by prominent blood vessels of different sizes surrounded by whorls of neoplastic meningothelial cells [26]. In case of incisional biopsy or if the lesion is not clearly defined in terms of location, another differential diagnosis could be vertebral angiomatosis. This entity has been described in one dog and is characterized by the proliferation of a mixture of arterial and venous vessels intermingled with small capillaries infiltrating the bone trabeculae and soft tissues [27]. The relative location to the spinal structures, vascular morphology, immunophenotype, neuroimaging features, and prognosis of all these conditions are summarized in Table 5.

For peripheral nerve HB, gross differential diagnosis included moderately invasive tumors, such as schwannoma, low-grade malignant nerve sheath tumor [33], and lymphoma [34], when accompanied by hemorrhage or intense vascularization. However, all these entities exhibit distinctive histological features.

The stromal cells of canine HB strongly expressed NSE and, less consistently, GFAP [7,8,9,10,11,13]. The latter marker may also be expressed by reactive astrocytes entrapped in infiltrating intraparenchymal tumors [35]. Vimentin has been applied in a few cases, yielding a positive reaction [7,10]. Stromal cells in human HB are immunolabeled with additional markers, including INHα, S100 protein [21], carbonic anhydrase IX [36] and XII, and brachyury [37]. INHα helps distinguish HB from metastatic clear cell renal cell carcinoma, which can have a similar microscopic appearance and develop in patients with VHL syndrome [38]. Furthermore, stromal cells are negative for epithelial markers like cytokeratin, which is typically found in clear cell renal cell carcinoma. In one study, the pattern of strong, diffuse expression of CAIX in combination with cytokeratin negativity was considered diagnostic for HB [36]. Stromal cells lack endothelial markers (i.e., vWF, CD31, and CD34), which highlight the tumor’s vascular network. All canine HBs in our series and one previously published case [10] were negative for INHα, whereas immunoreactivity was obtained in the control tissue (canine ovary). Conversely, CAIX immunoreactivity was observed in all tumors of our series.

The histogenesis of the stromal cells remains uncertain, but it has been shown that the microscopic precursor lesions occur mainly in the dorsal nerve roots [39] and in the molecular layer of the cerebellum [40]. The cells of origin are probably developmentally arrested hemangioblast precursor cells [41]. Studies on sporadic and VHL-associated HBs have found that loss or inactivation of the VHL gene leads to the accumulation of hypoxia-inducible factors (HIFs), particularly HIF1α, in stromal cells, which in turn triggers increased transcription of HIF1α-regulated genes, including those encoding VEGF, erythropoietin, and growth factors [1,21]. This pathway may explain increased angiogenesis and conditions conducive to neoplastic growth.

Spinal cord HB, when identified early and deemed accessible, may be surgically resected, and the prognosis is good with clinical improvement and long-term disease control. In the single case of excised forebrain HB [9], clinical deterioration was rapid, and the dog was euthanized two weeks following discharge due to poor neurological conditions, likely resulting from post-surgical complications. Stereotactic radiosurgery has proven to represent a reasonably effective and safe treatment option for CNS HBs in humans [42]. Continued collection of canine cases, coupled with advanced imaging and neurosurgical techniques, will be essential to refine prognostic expectations and optimize management strategies for this rare tumor.

## 5. Conclusions

HB is a rare primary tumor of the CNS, most commonly reported in the cervical and thoracolumbar spinal cord of adult dogs and may appear to be either intra- or extraparenchymal. There is no gender prevalence, and no specific dog breed is overrepresented. The tumor typically consists of two distinct cell populations (stromal and endothelial cells) and is comparable to the reticular subtype recognized in human HB. In dogs, useful markers are NSE and CAIX, while vimentin highlights the endothelial component as well as the stromal cells. INHα is likely not expressed in canine HB. Surgical resection may be the treatment of choice for both spinal cord and peripheral nerve HB.

This is the first reported case of peripheral nerve HB and its successful surgical treatment. Therefore, it is suggested to include this type of tumor, although rare, among the primary tumors of the canine peripheral nervous system.

## Figures and Tables

**Table 1:** Details of the primary antibodies.

Antibody	Clone	Dilution	Manufacturer
NSE	NSE-P1	1:200	Sigma-Aldrich, St. Louis, MO, USA
vWF	polyclonal	1:500	Sigma-Aldrich, St. Louis, MO, USA
VIM	V9	1:200	Dako, Carpinteria, CA, USA
GFAP	polyclonal	1:200	Dako, Carpinteria, CA, USA
INHα	R1	prediluted	Dako, Carpinteria, CA, USA
CAIX	polyclonal	1:500	GeneTex U.S., Irvine, CA, USA
Ki-67	MIB-1	1:200	Dako, Carpinteria, CA, USA

**Table 2:** Signalment, location, origin, and outcome of 6 cases of canine hemangioblastoma.

Dog	Breed	Gender	Age (y)	Location	Origin	Outcome
1	Boxer	M	8	T9-T10 intramedullary	PM	---
2	Mixed breed	nM	6	C6-C7 intramedullary	SB	no recurrence after 3 years
3	Beagle	F	10	C2-C3 intradural-extramedullary	SB	no recurrence
4	Labrador Retriever	nF	9	T12 intradural-extramedullary	SB	recurrence after 2 years
5	Yorkshire Terrier	nF	11	T11 intradural-extramedullary	SB	no recurrence after 3 years
6	Labrador Retriever	M	8	right sciatic nerve	SB	no recurrence after 18 months

nM = neutered male; nF = neutered female; PM = post-mortem; SB = surgical biopsy.

**Table 3:** Histomorphology of 6 cases of canine hemangioblastoma.

Dog	Cellularity	Capillary Component	Inflammatory Component	Vacuolization	Desmoplasia
1	++	+++	+	+	++
2	++	+++	+++	+	+++
3	+	++	++	-	+++
4	++	+++	+++	++	+
5	++	++	++	-	+
6	++	+++	+++	-	++

- = absent; + = mild; ++ = moderate; +++ = marked.

**Table 4:** Signalment, tumor location, and outcome of the published cases of canine hemangioblastoma.

Breed	Gender	Age (y)	Location	Outcome	Reference
Pointer	M	6	C5 and T5	euthanized	[14] *
Australian shepherd	nF	7	C4-C5	surgical resection	
NR	M	8	C7 intramedullary	euthanized	[8]
Pointer	M	6	T1 intramedullary	euthanized	[7]
Cross breed	NR	9	right olfactory bulb to frontal lobe	euthanized 2 weeks after surgical resection	[9]
Cross breed	nF	8	C1-C2 intradural extramedullary with paraspinal extension	no recurrence after 1 year	[10]
Yorkshire terrier	M	2	L3-L4 intramedullary	no recurrence after 9 months	[11]
Standard Poodle	F	3	T8 intramedullary	euthanized	[12]
American Pitbull Terrier	nM	3	brainstem intra-axial mass	euthanized	[13]

NR = not reported; nM = neutered male; nF = neutered female. * These 2 cases are included because of their characteristic histologic pattern of HB, although they were originally diagnosed as hemangioma [7,8,9,15].

**Table 5:** Differential diagnoses of spinal cord hemangioblastoma in dogs.

Lesion	Location	Vascular Morphology	Immunophenotype	Neuroimaging Features	Prognosis
Hemangioma [23,28,29]	Intramedullary, cervical and thoracic	Numerous dilated, tightly packed, thin-walled vascular channels without intervening tissue	Endothelial cells immunoreactive with vWF; smooth muscle actin (SMA) highlights pericytes	Capillary hemangioma: hyperintense on T2WI and mildly hyperintense on T1WI with strong contrast enhancement. Cavernous hemangioma: target-like appearance in both T1WI and T2WI	Improvement of clinical signs and no evidence of recurrence after surgery
Vascular hamartoma [14,30]	Intramedullary, thoracic and lumbar	Proliferation of thick-walled vessels of varying caliber without intervening neuroparenchyma	No unique profile: endothelial cells may be labeled with vWF or CD31; pericytes SMA-positive	Focal hypointense on T2W sagittal images. Marked contrast enhancement on T1W transverse images	Favorable if completely resected; poor if severe compression or untreated
Meningioangiomatosis [15,24,25]	Leptomeninges, intramedullary cervical and thoracolumbar	Perivascular spread of a mixed population of meningothelial and fibroblastic cells invading the nervous tissue from the leptomeninges	VIM-positive spindloid cells, negative for vWF, GFAP, S-100; endothelial cells labeled with vWF, CD31; occasionally SMA-positive pericytes	Mixed T2WI signal with the hypointense center coinciding with collagen deposition; mild T1WI hyperintensity with strong contrast enhancement	Generally poor, but surgical excision can reduce clinical signs and may be curative
Angiomatous meningioma [26,31,32]	Intradural extramedullary	Prominent blood vessels of different sizes surrounded by whorls of neoplastic meningothelial cells	Neoplastic cells immunoreactive with VIM, CD34 and E-cadherin	Hyperintense on T1WI with strong homogeneous post-contrast enhancement and dural tail	WHO Grade I; good prognosis after total resection; surgery challenging due to vascularity and edema
Vertebral angiomatosis [27]	Extradural, thoracic	Non-neoplastic vasoproliferative disorder within the vertebral bone and surrounding soft tissue	Endothelial cells diffusely and strongly immunoreactive with CD31 and vWF. The cells surrounding the capillaries are negative for NSE	CT: hyperdense lesion with a honeycomb appearance causing spinal cord compression. MRI: extradural lesion, hyperintense on T1W, T2W, and STIR images, with mild and irregular contrast enhancement	Rapid improvement after surgery; clinical signs relapsed 5 months later

## Data Availability

Data are contained within the article and Supplementary Materials.

## Supplementary Materials

The following supporting information can be downloaded at: https://www.mdpi.com/article/10.3390/ani15203010/s1, Figure S1: Canine ovary. The follicular cells consistently express INHα (INHα IHC; bar = 500 µm); Video S1: Resection of the tumor in dog 2.
