# Supplementary figures and images for: Canine Hemangioblastoma: Case Series and Literature Review
Source: Animals (Basel). 2025 Oct 16;15(20):3010. doi: 10.3390/ani15203010 (PMC12560891; doi:10.3390/ani15203010)

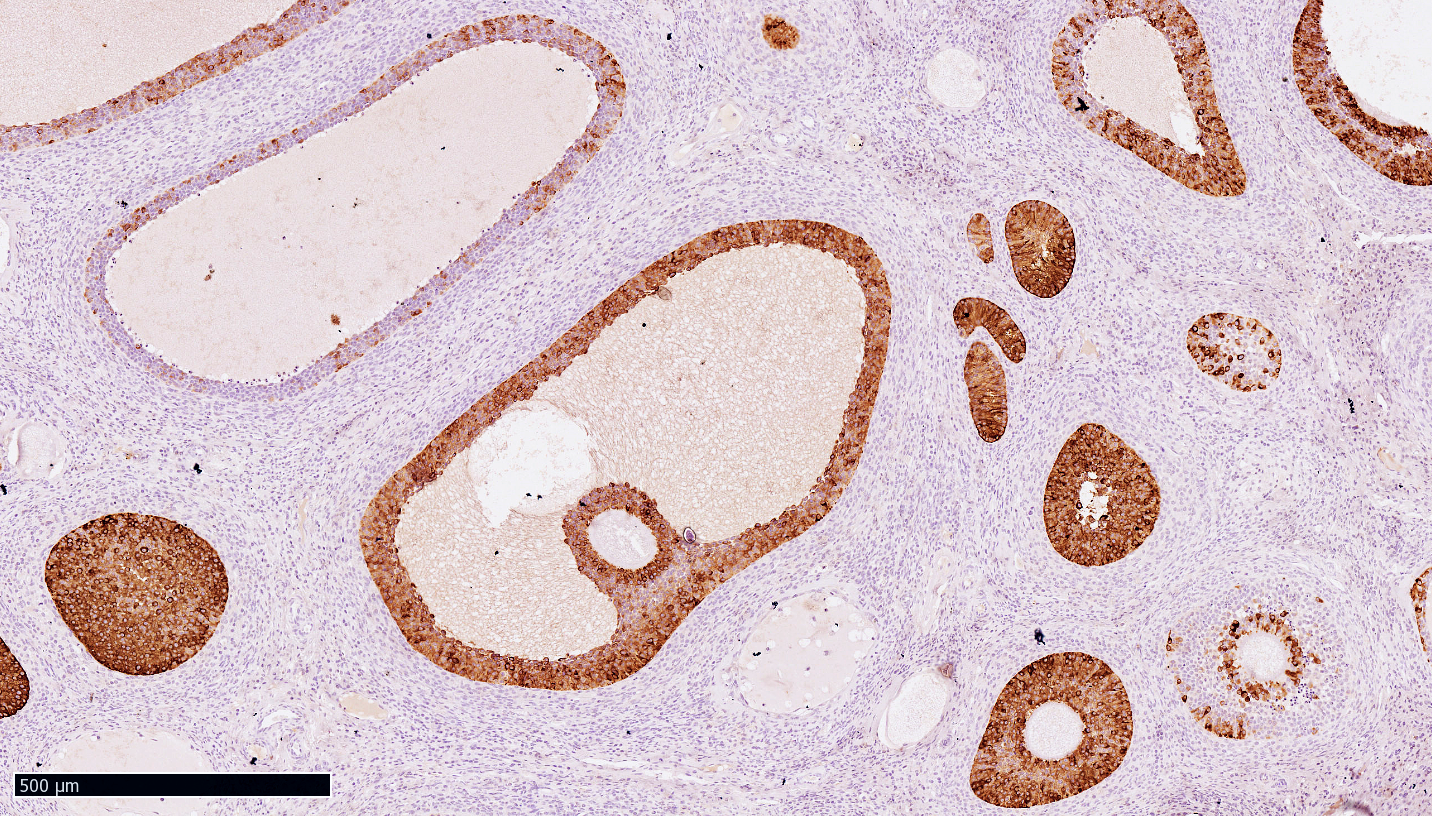

Supplement: Supplementary file 1 [file animals-15-03010-s001.zip › animals-3916762-supplementary/16489 cntr INHa 60x.tif]
